# Ashamed and Afraid: A Scoping Review of the Role of Shame in Post-Traumatic Stress Disorder (PTSD)

**DOI:** 10.3390/jcm5110094

**Published:** 2016-11-01

**Authors:** Tanya Saraiya, Teresa Lopez-Castro

**Affiliations:** 1 Gordon F. Derner Institute for Advanced Psychological Studies, Adelphi University, Garden City, NY 11530, USA; tanyasaraiya@mail.adelphi.edu; 2 The City College of New York, City University of New York, New York, NY 10031, USA

**Keywords:** shame, humiliation, embarrassment, trauma, PTSD, posttraumatic stress disorder, scoping review

## Abstract

Background: Despite considerable progress in the treatment of post-traumatic stress disorder (PTSD), a large percentage of individuals remain symptomatic following gold-standard therapies. One route to improving care is examining affective disturbances that involve other emotions beyond fear and threat. A growing body of research has implicated shame in PTSD’s development and course, although to date no review of this specific literature exists. This scoping review investigated the link between shame and PTSD and sought to identify research gaps. Methods: A systematic database search of PubMed, PsycInfo, Embase, Cochrane, and CINAHL was conducted to find original quantitative research related to shame and PTSD. Results: Forty-seven studies met inclusion criteria. Review found substantial support for an association between shame and PTSD as well as preliminary evidence suggesting its utility as a treatment target. Several design limitations and under-investigated areas were recognized, including the need for a multimodal assessment of shame and more longitudinal and treatment-focused research. Conclusion: This review provides crucial synthesis of research to date, highlighting the prominence of shame in PTSD, and its likely relevance in successful treatment outcomes. The present review serves as a guide to future work into this critical area of study.

## 1. Introduction

Post-traumatic stress disorder (PTSD) is a serious psychiatric condition associated with a substantial degree of life dysfunction, disability, and morbidity [1]. Although empirically validated therapies exist, a large proportion (30%–50%) of PTSD sufferers remain symptomatic following treatment [2]. Moreover, the significant symptom heterogeneity and comorbidities associated with PTSD continue to stifle therapeutic gains. Efforts to optimize treatment entry, continuity, and outcomes have led clinical researchers to expand investigations beyond the historically favored fear-based conceptualization and targets of the disorder. Models that implicate other negative and dysregulating affective experiences in PTSD’s development and maintenance have surged in popularity and relevance [3,4]. A likely reflection of this shift is the DSM 5’s re-classification of PTSD within its own chapter, apart from anxiety disorders, and the inclusion of negative alterations in cognition and mood as a distinct symptom cluster.

One of the negative affects garnering considerable attention in relation to PTSD etiology and course is shame—a common emotional reaction after traumatic exposure with extensive roots in both the theoretical [5] and clinical PTSD literature [6]. A universal and social emotion, shame can be briefly defined as a painful set of affective and cognitive states typified by self-judgment stemming from a perceived transgression of social/cultural norms or expectations. Although frequently interconnected, guilt is the negative evaluation of a specific behavior (“I’ve *done* an awful thing”) whereas shame condemns the self (“I *am* an awful person”) [7]. Their difference is further highlighted in what each emotion motivates: guilt may prompt reparative efforts whereas shame may trigger avoidance and withdrawal. While several theoretical models [8,9,10,11] differ on when shame occurs in the sequence of pre-trauma events to post-trauma, they converge on the functional role of shame in PTSD development. Experiencing shame, whether through attributing blame to the self or for one’s symptom presentation after diagnosis with PTSD, prohibits integration of the traumatic memory into one’s identity, thereby preventing recovery. Shame’s pathogenic impact appears particularly salient in the wake of chronic interpersonal violence due to the social subordination, powerlessness, and lack of control commonly experienced in these relational and traumatic occurrences [12]. Many have proposed that the loss of wholeness, integrity, and humiliation experienced in relational trauma is also more likely to generate intense feelings of shame rather than fear [11,13,14,15]. Evidence corroborates this finding: for a subset of individuals with PTSD, shame is *the* primary response to traumatic experiences [3]. Furthermore, shame after trauma has been associated with biological responses, suggestive of an innate reaction to social degradation in traumatic situations that can elicit the cardinal biopsychosocial symptoms of PTSD [10]. Lastly, ameliorating shame may be as critical as addressing fear to the recovery of a significant subpopulation of PTSD sufferers [16,17,18].

Given the increasing consensus that shame may be central to the etiology of PTSD as well as its prognosis and treatment, a review of the existing empirical support for shame’s relationship to PTSD is not only timely, but also imperative. In contrast to the numerous reviews of conceptual and clinical understandings of trauma and shame [10,11,13,14,18,19,20,21,22], no such exhaustive review of empirical research has been conducted. Most prior work has collapsed conceptual differences between shame and guilt, but some recent conceptual reviews have underscored the independent relationships shame and guilt have in PTSD [10,11,14]. Despite these advances, what is known about solely shame and PTSD in the empirical literature still remains unclear. Thus, in order to summarize and assist in the dissemination of relevant findings, we embarked upon a scoping review of the available empirical research on the relationship between shame and PTSD’s development, course, and treatment. With the overarching goal of presenting the general state of research activity, we employed a scoping review methodology as a means of capturing research spanning a variety of study designs and populations. We sought to synthesize the breadth of empirical research linking shame to PTSD, consider its implications for theory and practice, and identify the knowledge gaps that may serve as guideposts for future research.

## 2. Experimental Section

The present study followed the methodological framework of a scoping review as outlined by Arksey and O’Malley [23] to identify published studies investigating the relationship of shame to PTSD.

### 2.1. Search Strategy

**Figure 1:**
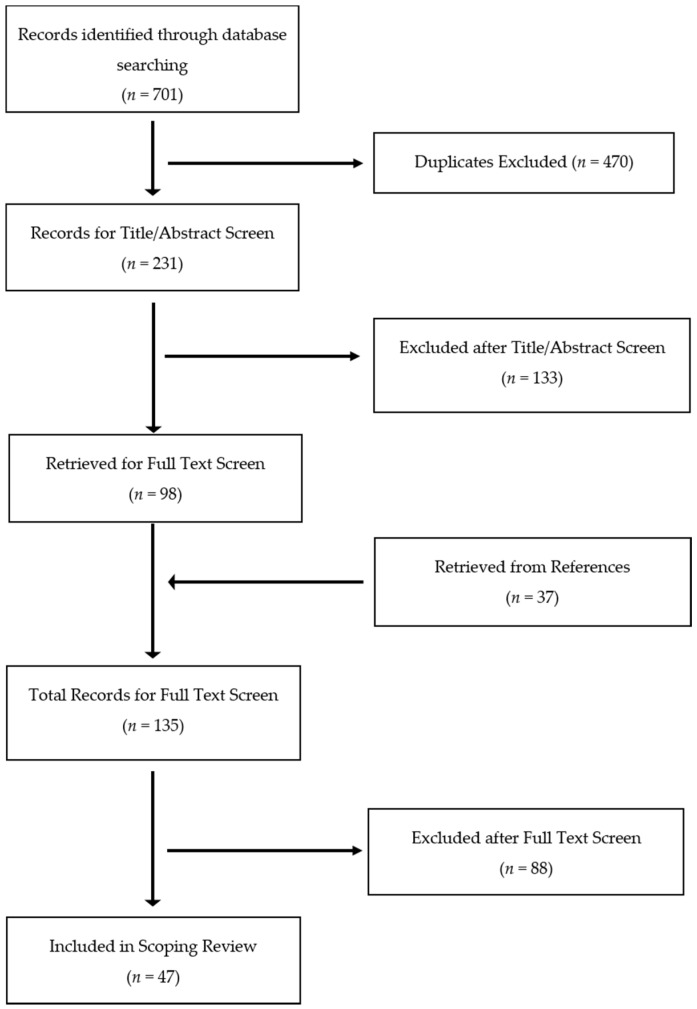
Flow diagram of scoping review article selection.

On the basis of prior scoping reviews [24,25], the databases PubMed, PsycInfo, Embase, Cochrane, and CINAHL were searched. For shame, the following search terms were used: *shame*, humiliation, embarrass*, disgrace*. To limit this scoping review to research specifically assessing and discussing shame, the search term *guilt* was not included. For PTSD, search terms included: *PTSD, PTSS* (post-traumatic stress symptoms)*, post-traumatic stress disorder, posttraumatic stress disorder, post-traumatic stress, post traumatic stress, and posttraumatic stress*. No date restriction was placed on the initial search. Screening of articles was conducted by two independent raters. After removal of duplicates, all search results were assessed for eligibility through a preliminary title/abstract screen and a subsequent full text review. References of eligible articles were reviewed for studies not identified by original search terminology. The number of articles reviewed at each stage in the search process are presented in Figure 1.

### 2.2. Inclusion and Exclusion Criteria

To be included, articles met the following criteria: (a) product of original quantitative research published in a peer-reviewed journal; (b) written in English (c) included a quantitative measure of shame (d) included a quantitative measure of PTSD and (e) identified shame as a primary variable of interest in relation to posttraumatic distress. Due to the scoping nature of this review and our interest in shame’s implication in the development of PTSD, PTSD diagnosis itself was not an inclusion criterion; investigations of PTSD symptoms in nonclinical samples as well as studies of samples without full PTSD were included. Since addressing the confounding of shame with other emotional responses was an aim of this review, studies were excluded if shame was assessed in aggregate with other negative emotions such as guilt, disgust, and fear. Similarly, studies that did not distinguish PTSD from other psychiatric sequelae of trauma were not included. Furthermore, since the aim of this scoping review was to focus on empirical research, case reports were excluded from the review.

## 3. Results

### 3.1. Overview of Results

In sum, 47 studies were included (see Table 1) with a total of 6642 participants.

The large majority of studies (*n* = 33) consisted of adult civilian samples. Far fewer studies examined children/adolescents (*n* = 7) and military personnel (*n* = 4). Three studies contained both adolescent and adult participants [26,27,28]. Both women and men were well represented. Representation of racial and ethnic minorities, however, was sparse and relegated to cross-cultural validation studies [29,30] or refugees [31]. Most employed a retrospective, cross-sectional design with thirteen studies reporting on longitudinal data [30,32,33,34,35,36,37,38,39,40,41,42,43].

Researchers relied almost exclusively on self-report measures to assess the construct of shame, representing an important limitation of the current empirical literature. Of the self-report scales utilized, the most frequently employed measures were the Test of Self-Conscious Affect (TOSCA) [44] (*n* = 9), Internalized Shame Scale [45] (*n* = 5) and the Experience of Shame Scale [46] (*n* = 4). A portion (*n* = 12) designed scales or questions exclusively for their investigation. The notable exceptions to this self-report trend were three studies, two of which utilized laboratory-based paradigms to examine implicit shame processing [9,47], and one that measured nonverbal shame through coding of facial expressions [28]. Similarly, posttraumatic stress symptoms were routinely measured with self-report questionnaires such as the PTSD-Checklist [48].

In terms of the construct of shame, the present review found significant diversity in its definition and operationalization across studies. Shame was examined as a trait (shame-proneness, generalized shame), as a product of traumatic exposure (trauma-related shame) and as state shame (specific to a task or time). A subset studied how shame-proneness and trauma-related shame converged, diverged, or interacted following trauma exposure [47,49,50]. Despite an association between the perpetration of war-related violence and PTSD [51], only one study on perpetration-related shame and PTSD met study inclusion criteria [52].

### 3.2. Types of Trauma Exposure and Shame

The studies reviewed suggest that the type of traumatic occurrence significantly influences the likelihood of shame. Shame was found to be greater in individuals surviving sexual violence compared to nonsexual interpersonal violence [53,54], in PTSS/PTSD patients with more than one traumatic exposure [31,54,55], and in cases where intimate partner violence (IPV) entails emotional abuse [56]. This is in line with theoretical models [14,57] where the emotional consequences of relational trauma and repeated traumas are linked with shame.

### 3.3. The Association of Shame with PTSD

The majority of the studies selected for review (*n* = 32) found significant associations between shame and PTSS/PTSD severity. Three of these studies were the first empirical investigations of shame in traumatized samples, and thus pivotal in establishing shame as a common affective experience in PTSD [6,58,59]. More recent research expanded upon this finding, investigating shame’s relationship with PTSD symptoms. Shame was significantly associated with greater negative and self-critical thinking [60,61], hyperarousal [36,38,62], avoidance [9,38,62], intrusive recollections [38], bodily shame [63,64], and negative attributions of the traumatic occurrence [37].

Studies employed various designs, construct definitions, and modes of assessments (e.g., self-report, physiological, and observational measures) to document associations between the multidimensional construct of shame and PTSD. For instance, shame was found to be associated with the biological stress response marked by increasing sympathetic nervous system activity [65], in addition to the disclosure of abuse [28] and with complex PTSD symptoms [66]. Yet, despite the heterogeneity of construct definitions and methodologies, the bulk of studies found shame to be significantly related to PTSD, providing relative convergence within the literature.

Some exceptions, however, were notable. For instance, a current state measure of shame, but not trait shame, was positively correlated with PTSS severity in a study of undergraduates with elevated PTSS [49]. Another study provided novel insights into the potential for discrepancies between the conscious report of shame and shame-related cognitive processes occurring outside awareness. Bockers et al. [47] collected data on both explicit and implicit shame, the latter with the Implicit Associations Test (IAT) [67]. The IAT measures implicit associations between target words (*self* vs. *other*) and attribute words (*shame/guilt* and *contentment*). In a series of blocks, words are presented and the participant must quickly categorize words as belonging to one of two groups. For instance, the word *embarrassment* may appear on screen, and the participant must categorize it as *self* or as *other* through different keyboard clicks. Faster reaction times denote stronger associations. In line with prior research, Bockers et al. [47] found that women with PTSD self-reported greater levels of shame than both trauma-exposed women without PTSD and a non-traumatized group of women. However, the study found no difference between the trauma-exposed and PTSD women in implicit shame-proneness, both showing significantly higher levels than non-traumatized women. Authors note that this similarity in implicit shame-proneness in the trauma-exposed groups may be related to their shared psychiatric diagnoses, since both groups were recruited from a psychiatric inpatient population.

### 3.4. Shame in the Development and Maintenance of PTSS and PTSD

Correlational research demonstrates shame as a likely component of PTSD, but it precludes conceptual understanding of *how* shame is implicated in PTSD. Relatively fewer studies (*n* = 10) examined the role of shame in the development of PTSS through either longitudinal designs or mediational analyses. Four longitudinal studies found shame to be a significant predictor of PTSS [32,42], poorer adjustment [37], and symptoms of hyperarousal, avoidance, and intrusive recollections [38] during follow-up assessment. Two of four mediational studies also found shame to be a significant mediator between childhood abuse and PTSS six months after a crime-related trauma [32] and between both emotional/verbal abuse and characteristics of dominance/isolation in interpersonal violence and later PTSD symptoms [68]. In sum, studies demonstrated that shame predicted immediate posttraumatic stress (PTS) reactions to a recent traumatic event, such as one to six months after a trauma [32,68], and future reactions to a traumatic event, such as one to six years afterwards [38].

Immediate reactions to a traumatic event appear critical to PTSD onset. Two studies found the immediate experience of a traumatic event as shameful, termed *peritraumatic shame* or *event-related shame*, to act as a mediator of PTSD symptom presentation. Peritraumatic shame was a mediator between both interpersonal traumatic exposure and greater traumatic exposure with later PTSD symptoms [54], and event-related shame was a mediator between shame-proneness, a personality trait variable, and PTSS [50]. These studies suggest immediate experiences of shame after a traumatic event elicit PTSS, given pre-existing risk factors. One of these pre-existing risk factors is based on individual propensities to experience shame [50]. Indeed, two other studies corroborated this finding. Shame was a significant mediator between self-blaming internal attribution styles and PTSD symptoms immediately after abuse and one year after abuse [29,36]. Thus, as a whole, these studies suggest that both personal inclinations to experience shame in addition to peritraumatic shame mediate PTSD onset both directly after a traumatic event and over time.

If shame is a risk factor of PTSD, the question remains as to whether shame maintains posttraumatic symptoms. Results from one psychotherapy process study strongly implicated shame as contributing to the maintenance of PTSD. In measurements of week-to-week levels during treatment, increases in trauma-related shame were associated with subsequent increases in PTSD symptoms [42]. In contrast to this finding, a longitudinal study of Korean sexual assault survivors found that shame-proneness (one to four months post-trauma) did not predict PTSD levels at a second assessment one month after the first [30]. Research design variations such as the type of shame studied (general versus trauma-specific), proximity of the time points assessed (days, weeks or months), and sample characteristics may all directly impact how shame is understood to maintain PTSD. With data from only two studies meeting criteria for the current review, further research into this area is certainly warranted.

### 3.5. Shame as an Outcome and Mediator of PTSD Treatments

In proportion to the large number of studies linking shame to PTSD and its symptomatology, little empirical data is currently available on the role of shame within the PTSD treatment context. This represents the largest gap in the present literature. Empirical research findings on child interventions are limited to one randomized clinical trial comparing trauma-focused, cognitive behavioral therapy (TF-CBT) to child-centered therapy (CCT) for survivors of sexual abuse [34,35]. Children’s shame was measured by self-report at pre- and post-intervention and at 6 and 12 month follow-ups. Although both treatments demonstrated marked reductions in PTSD symptoms and trauma-related shame, children who received TF-CBT improved significantly more across all symptom domains, including shame. Notably, TF-CBT’s differential gains in shame alleviation were still present a year after treatment. However, no predictive relationship was found between children’s pre-treatment shame and either treatments’ outcomes. Authors suggested that TF-CBT’s attention to the gradual exposure to and processing of trauma-related memories may have been central to the reduction in shame-based cognitive distortions [34]. No studies testing this mechanism for shame reduction were found during the present review.

Of studies meeting inclusion criteria, four randomized clinical trials (RCTs) assessed shame as an outcome of PTSD treatment in adults [39,40,42,43]. The scarcity of empirical data on the role of shame within adult PTSD interventions was notable. Nevertheless, preliminary findings are promising. Decreases in shame were evident across all therapeutic approaches tested. Cognitive processing therapy (CPT) and its separate components (written accounts and cognitive therapy) [43], prolonged exposure (PE) and modified PE with image rescripting [42], and dialectical behavioral therapy (DBT) and DBT combined with PE [40] were each associated with significant changes in self-reported shame. Of particular interest given the social basis of shame, trauma-focused and present-focused group psychotherapy were both found to reduce shame [39]. Notably, a relative superiority of one treatment for shame reduction was not found. This is particularly significant when considering the results of two RCTs which compared a shame-targeting treatment to a PTSD treatment with no specific shame focus [40,42].

Although the necessity of formally and explicitly targeting shame for its reduction within PTSD treatment remains unclear, the limited literature to date has strongly demonstrated shame’s importance to recovery from PTSD. Evidence from two clinical trials suggests shame is critical to the change process in PTSD treatment. When compared to a waitlist control group, adult women survivors of CSA receiving six months of either trauma-focused or present-focused group psychotherapy reported significant reductions in shame [39]. Moreover, the active treatment conditions mitigated shame’s association to PTSD symptoms. In contrast, shame remained closely tied to the PTSD symptoms of women in the waitlist group. Mediational analyses from the same study revealed that changes in self-reported shame mediated treatment response, accounting for approximately a third of PTSD improvement. In further support of shame’s importance to treatment response, a Norwegian inpatient trial for treatment-resistant individuals found a temporal relationship between shame and subsequent PTSD improvement [42]. Collecting weekly in-treatment data, investigators employed a multi-level approach to disaggregate between-person and within-person effects of changes in trauma-related shame upon PTSD recovery. A shift in shame relative to the individual’s typical level predicted PTSD symptoms three days later. The reverse—increases in PTSD symptoms predicting increases in shame four days later—was not found. These findings present the possibility of a shame-centered mechanism of change within PTSD treatment.

Although not a clinical trial, one study contained initial support for specifically shame-reducing therapeutic strategies. Comprehensive distancing, an acceptance and commitment therapy (ACT) strategy, as well as a CBT-based cognitive restructuring exercise alleviated state shame in a non-treatment seeking undergraduate sample with elevated PTSS [49]. However, in contrast to findings from other clinical samples, trait shame was found to be unrelated to trauma symptomatology, raising doubts to the study’s generalizability to more distressed and treatment-oriented populations. Nonetheless, the study provides an initial template for future investigations on mechanisms by which shame is potentially transformed through treatment.

### 3.6. Nonsignificant Findings of Shame in PTSD

A small proportion of studies (*n* = 7) in the present review found no significant relationship between shame and PTSS/PTSD. Two of these studies found shame to be related to depression rather than PTSD [30,69] where one of these studies found guilt to be associated with PTSS instead of shame [69]. These two studies may lack significant associations between shame and PTSS/PTSD because of the traumatic exposures in the respective samples. For example, shame was significantly associated with PTSD in two samples of undergraduate women and community women with abuse and interpersonal violence histories, but was not significantly associated with community women with nonsexual intimate partner violent histories [53]. Similarly, shame was not significantly associated with reckless drivers with high risk for PTSD [52], suggesting that interpersonal traumatic exposure, and specifically sexual abuse, are more highly associated with shameful affect. This is consistent with theoretical models of social emotions [11,14,15,16].

### 3.7. Shame Distinguished from Guilt in PTSD

Within the reviewed studies, a substantial amount of interest has centered on clarifying the interconnected nature of shame, guilt, and PTSD. Close to half (*n* = 22) of included studies provided results in which shame and guilt were differentially assessed and evaluated. When compared, a majority found shame to have a more salient association with PTSD and PTSD severity than guilt [27,39,50,56,59,70,71]. These findings dovetail with theoretical models that hypothesize shame to be more toxic than guilt due in part to its condemnation of the self—versus guilt’s condemnation of an action or thought—and the subsequent increase in posttraumatic distress associated by negative self-concepts [72]. Several of the studies supporting this conclusion are notable for their efforts to control for guilt’s unique contribution to PTSD and related symptoms [70,71] and one for statistically accounting for the theoretical overlap between guilt and shame, termed *shame-free guilt* and *guilt-free shame* [50]. However, given that the most frequently employed scale for assessing guilt and shame, the TOSCA, has been criticized for its overvaluation of guilt’s positive outcomes [73,74], it is unclear how much measurement artifact has contributed to this line of findings. Nevertheless, the case for the relative centrality of shame over guilt in PTSD is also suggested by findings which implicate shame over guilt in mediating PTSD’s relationship to psychological abuse exposure [56,66] and negative outcomes such as aggression [75,76]. Moreover, in the treatment realm, clinically targeting and reducing shame may prove more instrumental than decreasing guilt. For women with histories of childhood sexual abuse, improvements in shame, not guilt, mediated the effect of two different PTSD treatments [39].

Expectedly, not at all studies reviewed converged on shame’s relative importance over guilt. Past-month experiences of shame and guilt were each independently associated with PTSD symptoms in survivors of Norway’s Ujola Island terrorist act [26]; similar findings were found in a sample of assaulted Korean women [30]. Both shame and guilt independently mediated the association between PTSD symptoms and suicidal ideation in an active military sample [77]. It is probable that a host of factors may contribute to the fluctuating primacy of shame over guilt in PTSD pathogenesis. Variations in trauma type, chronicity, and *pre-trauma* and *post-trauma* attribution style may raise or lower the importance of shame in relation to guilt for PTSD development and course. Divergent results also underscore the variety of construct definitions and the sheer complexity of comparing two multidimensional emotions functioning at varying levels of awareness [47].

Heterogeneity in PTSD symptom presentation is also worth considering when examining the relative strength of shame and guilt’s contribution to the disorder. Importantly, shame and guilt have been shown to have unique and varying associations to each PTSD subcluster [62]. As logic might suggest, shame was found to be more predictive than guilt of avoidance-related behaviors; likewise, guilt, and not shame, predicted re-experiencing symptoms. Both guilt and shame were jointly linked to hyperarousal difficulties. So, it is likely that an individual’s distinct constellation of PTSD symptoms may dictate how prominent a role shame or guilt will assume.

Acknowledging the clinical proximity and interaction between guilt and shame, one study attempted to model the interrelatedness of guilt and shame in the context of PTSD. Held et al. [78] found support for two pathways linking trauma-related guilt to trauma-related shame. In addition to a direct association to shame, those with trauma-related guilt were more likely to employ avoidant coping styles characterized by cognitive and affective disengagement. Avoidant coping was in turn associated with trauma-related shame. Findings highlight the need to account for PTSD’s developmental course when considering the probable synergy between guilt and shame in PTSD.

### 3.8. Shame as a Mediator between PTSD and Negative Outcomes

A sub-group of studies (*n* = 5) have investigated how shame acts as a mediator between PTSD and subsequent emotional and behavioral responses. The majority of these studies found shame to mediate between PTSD and aggression [9,75,76,79], but one study also found shame to mediate between PTSD and suicidal ideation [77]. Three studies found shame to be a partial mediator or full mediator between PTSD and an aspect of aggression in primarily male veteran samples. Shame mediated PTSD and verbal aggression [75], aggressive behavior [76] or the likelihood of IPV perpetration [79]. Moreover, guilt was found to be an insignificant mediator between PTSD and aggression in two of these studies, increasing the likelihood that shame is uniquely associated with dire consequences in the aftermath of PTSD [75].

One particularly novel experimental paradigm tested how implicit shame primed in PTSD participants influenced IPV perpetration in both a male and female civilian sample [9]. PTSD participants processed shame faster than their non-PTSD counterparts and presented greater likelihood of IPV perpetration. The authors suggest that shame is a devaluing experience that places blame on the self and PTSD individuals may fall back on aggressive behaviors to restore self-worth, which only produces more shameful affect. Inadvertently, this pattern promotes a cycle of violence and shame. Most mediational studies in this review assessed a male Veteran sample, but Sippel and Marshall [9] examined civilian men and women, suggesting this cycle of violence can be seen across populations.

## 4. Discussion

The current study serves as the first review of the empirical literature on the role of shame in PTSD etiology, course, and treatment. Adopting a scoping review methodology, our primary aim was to organize and synthesize the extant quantitative research concerning shame and PTSD. Results from our review confirmed the presence of a substantial body of quantitative research on the association of shame to PTSD and the availability of a considerable amount of data supporting shame’s functional role in the disorder’s emergence and maintenance. Shame was consistently related to posttraumatic distress and symptomatology with evidence suggestive of a shame-based variant of PTSD. Although relatively understudied, the role of shame within PTSD treatment appeared noteworthy as intervening upon shame not only successfully reduced shame, but also corresponded with improvements in PTSD. Empirical research also confirmed that when the shared variance between guilt and shame is accounted for, shame remains the more pathogenic of the two, with guilt more likely to become problematic only when compounded with shame. Shame’s unique relationship with aggression was also evident in the reviewed studies, shedding explanatory light on how posttraumatic distress may act as kindling for future violence. Taken as a whole, the body of reviewed studies marks significant methodological progress made towards the study of—and consequently, strong evidence for—an association between shame and PTSD.

In addition to summarizing the available empirical evidence, a second study aim was pinpointing its design limitations. Exclusive employment of self-report measures and non-validated assessment tools stood as major limitations of the current body of quantitative research. Several studies which assessed implicit shame-related processes indicate that self-reported shame may be telling only one side of the story—and that the awareness of shame is very likely a distinguishable construct from shame processing per se. Review also revealed sparse empirical data investigating the impact of shame on PTSD treatment, and no data on shame-specific interventions for PTSD.

### 4.1. Implications of Research

Findings in this scoping review provide important implications for research on two theoretical models delineating the relationship between shame and PTSD. The first model, proposed by Feiring et al. [8] proposes traumatic exposure followed by negative appraisals and attributions of both the traumatic event and cues associated with the event lead to shame. In turn, the experience of shame increases the likelihood for PTSD onset. Our findings in this scoping review provide support for this pathway; both peritraumatic appraisals and self-blaming attributions have been found to generate shame, which then yields PTSD [29,36,53]. In fact, shameful peritraumatic appraisals may be uniquely tied with PTSD outcomes, whereas other peritraumatic appraisals (e.g., fear, betrayal, alienation, anger, and self-blame) are associated with a combination of PTSD and depressive outcomes [53]. Appraisals appear to generate shame through the traumatic individual’s interpretation of his or her thoughts, feelings, and behaviors in a self-denigrating style [53]. More specifically, the traumatized individual perceives his or her reactions to the traumatic event, posttraumatic symptoms, and perceptions of permanent alterations in the self as shameful and embarrassing, and the culmination of these appraisals lead to an attribution of self-blame [8,80].

The experience of such intense blame towards the self induces shame and withdrawing behavior. Indeed, shame has been associated with general PTSS symptoms, but appears to primarily lead to avoidance behaviors, a hallmark symptom of PTSD [32,37,38,62]. The inability to process shame and the discomfort with experiencing shame leads to a fear of shame itself; shame becomes an internal threat. In addition, shame is avoided because of its association with both the traumatic event and traumatic cues [11,14,16,29,80]. However, we cannot conclude that shame acts directly on avoidance behaviors, since contrary evidence suggests the combination of either shame, fear, and anger or shame and guilt leads to avoidance behaviors [62,78]. More research is needed to understand how shame may generate sub-threshold and full-PTSD, but what is clear is that the inability to process shame contributes to avoidance behaviors, which in turn may generate other symptoms of PTSD, namely psychic numbing and alexithymia [11,14,16].

A second model proposes shame develops after PTSD diagnosis or coincides with its development and leads to dysregulating affect and behaviors, primarily aggression [10,20,21,22]. Some of the studies in this review support this model, finding shame to be a significant mediator between PTSD and aggressive behaviors, even IPV perpetration [9,75,76,79]. Aggression may be so common in PTSD individuals with shame because of their sensitivity around their self-worth. Aggression can protect the vulnerable self from perceived rejection [9,10] and reduce judgment in the eyes of others [11] or alternatively, aggression can be due to projecting self-blame and self-hatred onto others [11,81] and a consequence of fewer cognitive resources and empathy for others [10].

This relationship between shame and aggression ultimately suggests that shame may be a cardinal variable maintaining cycles of violence, where PTSD individuals experiencing shame engage in violence that inevitably begets further shame and violence [9,76]. In future research, developing clinical treatments that address shame may have widespread effects on reducing repetitive violence exposure, especially important given the poorer prognosis associated with multiple trauma exposures [82]. Indeed, what appears salient in both of these models is that the inability to process shame—and by extension the traumatic event and traumatic cues—begets violence, PTSD, and avoidance.

The widespread use of self-report measures was a prominent feature of the current literature. This skew towards explicit shame has significant implications for research. The well-accepted limitations of self-report questionnaires are especially relevant when attempting to investigate aversive cognitive and affective states—of which shame is paramount. Self-report measures require the respondent to not only be conscious of the psychological state in question, but also be willing to acknowledge and disclose details of its experience. It is known that various factors associated with PTSD and traumatic stress may derail each of these requirements. For instance, individuals with PTSD are more prone to manage painful cognitions and emotions by using avoidance [83]. Moreover, chronic trauma exposure during critical developmental periods produces long-lasting deficits in emotion regulation including disruptions in the ability to accurately recognize and describe affective experiences [84]. Thus, for individuals with PTSD, shame may evade explicit awareness altogether, and instead, measurements of implicit shame may more accurately capture shame-related processes. For this reason, future research integrating explicit and implicit measures may provide not only a more accurate assessment, but also insight into the differences between explicit and implicit shame-related processes.

### 4.2. Implications for Clinical Practice

The present study carries significant implications for clinicians working with traumatized populations. The robustness of evidence in support of shame’s liability and toll underscores the clinical importance of assessing shame in patients’ histories, being attuned to shame states within the therapeutic encounter, and when appropriate, formulating shame-specific interventions and goals. In general, findings demonstrated a strong association between peritraumatic shame and the emergence and severity of PTSD. Thus, a clinician’s ability to locate and ameliorate trauma-related shame effectively is likely to have important repercussions for the disorder’s course. In terms of shame’s relevance to certain clients, results from IPV-centered research suggest that addressing shame in the treatment of PTSD related to chronic relational trauma is not only warranted but may be fundamental to recovery. In particular, research on shame’s mediating role between IPV exposure and PTSD suggests the singularity of psychotherapeutic interventions which can therapeutically examine shame’s impact upon symptoms and self-concept.

Importantly, the wealth of evidence relating shame to PTSD suggests that it likely stands as a potent treatment barrier and probable contributor to early drop-out and attrition. Appreciating the role of shame in patients’ lives may help clinicians proactively work with avoidant coping strategies and other shame-triggered responses which pose significant challenges to successful treatment outcomes and may be misinterpreted as purely fear-based disengagement. Relatedly, data from the reviewed studies noted a significant relationship between trait shame and aggression for PTSD sufferers, providing a possible mechanism for the well-known association between posttraumatic stress and perpetration of interpersonal violence [85]. For the clinician, these findings emphasize understanding of how shame-prone patients may attempt to regulate shame states following traumatic exposure and the clinical utility of linking aggressive behaviors with implicit shame processes in PTSD-related presentations. The reviewed research suggests that employing techniques or interventions that promote shame’s adaptive regulation [86] may readily assist in the reduction of critical treatment-interfering behaviors.

Review of the small yet encouraging domain of treatment literature indicates that reducing shame during PTSD intervention is clinically advantageous, not only in expressly attenuating shame but in affecting overall posttraumatic symptom changes. This is in line with the emerging support for compassion-oriented interventions [87] and self-compassion as a potent mechanism of change within psychotherapy for PTSD [88]. The current review found evidence that psychotherapy may help contain the toxic effects of shame, creating a buffer between experiences of shame and trauma-related distress [39]. Of interest to clinical practice, the current review contributes to the discourse on whether maladaptive social emotion processing can be effectively targeted with fear-modeled PTSD interventions. Review of the relevant treatment studies suggested multiple clinical pathways for reducing shame, which may or may not involve shame being directly targeted or processed. Of the adult treatment studies reviewed, CPT [43], modified PE with imagery rescripting [42], and DBT PE [40] were purported to intervene explicitly on dysfunctional shame and related cognitions. Reductions, though, in shame were also observed in other modalities such as traditional PE, standard DBT, group psychotherapy and narrative writing exercises. However, because of the relatively few studies examining how changes in shame impact PTSD treatment, questions regarding the preferential treatment selection for shame-centered PTSD remain largely unanswered.

Implications for the treatment of children suffering from trauma-related difficulties are derived from one RCT [34,35]. In this sample, focusing on the traumatic memory through gradual exposure and emotion processing was found to improve PTSD and shame self-concepts significantly more than coping skills and empowerment-orienting therapy. Indeed, study investigators anecdotally reported that children found the direct discussion of trauma to be the most helpful treatment component. However, conclusions that can be drawn are limited due to the severity and chronicity of the sample’s trauma histories. It is possible that other interventions, such as the nondirective CCT approach tested, may be sufficient for shame alleviation in less symptomatic child populations.

### 4.3. Limitations of this Scoping Review & Future Directions

Despite the gains of this scoping review, some limitations should be mentioned. The intention of a scoping review is to survey the breadth of literature available [23]. In doing so, they refrain from assessing the quality of studies. Many studies had important limitations, including the use of convenience samples, non-validated measures, and the lack of clear operational definitions. Furthermore, by focusing on shame, we did not systematically search overlapping constructs, such as guilt, which some literature may use synonymously with shame. We restricted our review to posttraumatic distress associated with PTSD, and thus we may have missed important research highlighting how peritraumatic shame may lead to other psychiatric problems such as major depressive disorder or substance use disorders. Lastly, our exclusion of qualitative data may have limited our ability to understand the diverse definitions and unique experiences of shame across individuals.

Overall, this scoping review presents the first summary of the state of empirical research on shame and PTSD. Given our findings, the next phase of scientific investigation should begin with a systematic review of a portion of the literature included in the current study (e.g., adults, correlational designs). A systematic review could also provide an evaluation of the quality of the research, a particularly important next step. Shame research must begin to consolidate construct definitions and utilize multimodal assessments (e.g., physiological and self-report) to adequately capture the multidimensional nature of shame. Furthermore, a large gap is the relative lack of studies investigating the nonverbal, physiological, and biological components of shame. To date, advances in technology, such as eye-tracking devices that can record gaze direction and attentional processes, have not been applied, despite the known connection between complex trauma, averted gaze, and emotion regulation deficits [19]. The neural correlates of shame processing in PTSD remain wholly uncharted. Investigation in these areas will provide a comprehensive understanding of how shame operates across social cognitive and neurobiological domains. In turn, clinical treatments targeting shame can be more precise at reducing this debasing affective experience and its impact on the debilitating symptoms of PTSD.

## Figures and Tables

**Table 1:** Characteristics and key findings of reviewed studies.

Author (Year)	Sample Type (*n*)	Design	PTSS/PTSD Measure ^2^	Shame Measure ^2^	Findings
Aakvaag et al. (2014)	Adolescent and adult survivors of terrorism (*n* = 325)	Cross-sectional	PTSD-RI	PTSD-RI (2 items)	Past month trauma-related shame and guilt associated with PTS ^1^ in mass-trauma survivors. Greater severity of trauma-related shame or guilt associated with greater PTS severity.
Andrews et al. (2000)	Adult victims of violent crime (*n* = 157)	Longitudinal	PSS-SR	Study-designed semi-structured interview (single item)	Post-traumatic shame and anger predicted PTS at 1-month after crime victimization. Post-traumatic shame and 1-month PTS predicted 6-months PTS. Post-traumatic shame mediated childhood abuse and 6-months PTS.
Badour et al. (2015)	Adult survivors of interpersonal trauma (*n* = 1522)	Cross-sectional	NSES	NSES	Peritraumatic anger, shame, and fear, in order, predicted assault related-PTSD (AR-PTSD). Peritraumatic shame was more associated with AR-PTSD in women; peritraumatic fear was more associated with AR-PTSD in men.
Barr (2011)	Couples with sick newborn infants (*n* = 67)	Cross-sectional	PCL-S	TOSCA	Shame-proneness predicted depression, but not PTS or anxiety. Guilt was a significant predictor of PTS symptoms, depression, and anxiety.
Beck et al. (2011)	Female IPV ^1^ survivors (*n* = 63)	Cross-sectional	CAPS	ISS	Trait shame, guilt-related distress, and guilt cognitions positively correlated with PTSD. Shame was positively correlated with higher levels of emotional and verbal abuse. Women with IPV marked by high dominance/isolation or emotional/verbal abuse and high shame had higher PTSD severity. Guilt was not a significant moderator.
Beck et al. (2015)	Female IPV survivors (*n* = 109)	Cross-sectional	CAPS	ISS	Trait shame and depression predicted negative thoughts of the self; shame had a larger effect size. Higher guilt associated with negative thoughts of the world. Higher levels of trait shame and guilt but lower levels of depression associated with greater self-blame.
Bennett et al. (2016)	Adolescent and young adults living with HIV (*n* = 88)	Cross-sectional	CPSS	TOSCA-A	Shame-proneness was positively associated with higher levels of PTSD and depression symptoms. Depression was predicted by shame-proneness and HIV-related stigma whereas PTSD was predicted by HIV-related stigma and avoidant coping.
Bockers et al. (2015)	Inpatient sample of female survivors of interpersonal trauma: with PTSD (*n* = 28), without PTSD (*n* = 32), non-traumatized (*n* = 92)	Cross-sectional	MINI	TOSCA and IAT (implicit)	Explicit shame and guilt were significantly different across groups; PTSD group had the highest and the non-traumatized group had the lowest. No differences in implicit shame between PTSD group and traumatized/no PTSD group.
Brewin et al. (2000)	Adult victims of violent crime (*n* = 138)	Longitudinal	PSS-SR	Semi-structured interview (single item)	Trauma-related shame experienced by a subset of participants and significant predictor of PTSD.
Bryan et al. (2013)	Active duty military personnel (*n* = 69)	Cross-sectional	PCL	PFQ2	Guilt and trait shame are higher in military personnel with history of suicidal ideation. Both guilt and trait shame mediated the relationship between PTSD or depression and suicidal ideation; guilt had a stronger relationship with suicidal ideation.
Cohen et al. (2004)	Sexually abused children (*n* = 203)	Longitudinal; RCT ^1^	K-SADS-PL	CAPS	Children randomized to TF-CBT showed greater reductions in shame attributions and PTSD from baseline to post-treatment in comparison to children in CCT ^1^.
Crocker et al. (2016)	Returning veterans, primarily male (*n* = 127)	Cross-sectional	PCL-S	ISS	PTSD predicted trait shame and global guilt. When controlling for global guilt, shame partially mediated PTSD symptoms and verbal aggression.
Dahl (1989)	Young adult and adult survivors of rape and attempted rape, primarily female (*n* = 55)	Cross-sectional	IES and semi-structured interview	CPRS (single item)	Assault-related shame, guilt, and suicidal ideation present in the majority of rape survivors.
Deblinger et al. (2006)	Child survivors of sexual abuse (*n* = 183)	Longitudinal; RCT	K-SADS-PL	TSQ and CAPS	Children in TF-CBT ^1^ showed significant decrease in trauma-related shame during post-treatment, 6-month, and 12-month follow ups in comparison to children in Child-Centered Therapy.
DePrince et al. (2011)	College students with at least one traumatic event (*n* = 98); Adult female survivors of child abuse/interpersonal crime (*n* = 94); Adult female survivors of nonsexual IPV (*n* = 236)	Cross-sectional	RCMS and PDS	TAQ	Posttraumatic shameful appraisals predicted PTSD in college students and female survivors of child abuse or interpersonal crime. Posttraumatic shameful appraisals did not predict PTSD in the nonsexual IPV sample.
Dewey et al. (2014)	College students (*n* = 144)	Cross-sectional	PCL-S	TEQ (subscales: anger, fear, guilt, shame, disgust)	In order, peritraumatic fear, anger, and shame were the top three predictors of avoidance and numbing symptoms. Peritraumatic guilt, fear, and anger predicted re-experiencing; shame did not. Peritraumatic guilt and shame were the strongest predictors of hyperarousal symptoms.
Dorahy et al. (2013)	Treatment-receiving adult survivors of Northern Irish conflict exposure with PTSD (*n* = 65)	Cross-sectional	SRC and clinical interview	CoSS, SSGS, and PFQ-2	Clinical dissociation group had significantly higher levels of (a) trait shame and guilt and (b) state shame, guilt, and pride than the subclinical dissociation group. Dissociation, trait/state shame, and trait/state guilt significantly predicted complex PTSD.
Dorahy et al. (2016)	Adults with Dissociative Disorders (*n* = 39); Two comparison groups: adults with child abuse-related chronic PTSD (*n* = 13) & adults with mixed psychiatric diagnoses and child abuse histories (*n* = 21)	Cross-sectional	CTQ and clinical interview	PFQ-2	Child maltreatment significantly contributed to trait shame and child abuse and neglect significantly contributed to trait guilt. Emotional abuse was the strongest predictor of both trait shame and trait guilt.
Dyer et al. (2015)	Adult female German inpatients/outpatients with PTSD from CSA ^1^ (*n* = 23), BPD (*n* = 25), BPD and PTSD after CSA (*n* = 22), and healthy controls (*n* = 27)	Cross-sectional	SCID-I	SBA and BIGSS	Participants with child sexual abuse identified more body areas as associated with traumatic experiences. Psychiatrically diagnosed groups rated trauma-related body areas to have more body-related shame, guilt, and disgust compared to the healthy controls.
Feiring et al. (2002)	Child and adolescent survivors of CSA (*n* = 137)	Longitudinal	IAE and CITES-R	Study-designed measure	Trauma-related shame was a significant mediator between abuse attributions both after the traumatic event and one year later with both PTSD and depression symptoms.
Feiring et al. (2002)	Child and adolescent survivors of CSA (*n* = 147)	Longitudinal	CITES-R and TSI	Study-designed measure	Trauma-related shame at abuse discovery was a small predictor of PTSD, depression, and self-esteem, but trauma-related shame one year after abuse was a strong predictor of PTSD, depression, and low self-esteem. Girls showed more shame than boys at the time of abuse, but decreased in shame over a year. Boys did not show a significant decrease in shame from abuse discovery to one year later.
Feiring and Taska (2005)	Adolescent survivors of CSA (*n* = 118)	Longitudinal	CITES-R and TSI	TOSCA and study-designed measure	Trauma-related shame experienced one year after CSA predicted high trauma-related shame six years after CSA and symptoms of hyperarousal, intrusive recollections, and avoidance. Children low in trauma-related shame have better treatment prognosis.
Freed and D’Andrea (2015)	Adult female survivors of interpersonal violence with PTSD (*n* = 27)	Cross-sectional	PCL-IV	PANAS (single-item) and study-designed adjective list	Shame-proneness was the only predictor of autonomic arousal in a trauma reminder task. Inactivation of the peripheral nervous system in PTSD patients was associated with fear and shame at baseline, anxiety and shame during the task, and shame during recovery period. Trait shame predicted lower respiratory sinus arrhythmia during recovery suggesting difficulty regulating affect after trauma reminders. State shame was the only predictor of lower RSA during the task, more than fear and anxiety.
Ginzburg et al. (2009)	Adult female CSA survivors (*n* = 166)	Longitudinal; RCT	PCL-S	ARBQ (Shame subscale)	Both treatment conditions demonstrated reductions in PTSD, abuse-related shame, and guilt. Improvement in shame mediated treatment effect on PTSD. No mediating effect found for guilt.
Hagenaars et al. (2011)	Treatment-seeking adults (*n* = 110)	Cross-sectional	CAPS	Study-designed single item	Multiple trauma group reported more recent shame experiences than single trauma group, independent of PTSD severity. Association between shame and childhood versus adult trauma group did not hold after controlling for PTSD severity.
Harman and Lee (2010)	Treatment-seeking adults (*n* = 49)	Cross-sectional	PDS	ESS	Shame-proneness positively correlated with self-criticizing thinking style, above and beyond contributions of PTSD and depressive symptoms.
Harned et al. (2014)	Adult females with comorbid BPD ^1^ (*n* = 26)	Longitudinal; RCT	PSS-I	ESS	Clinically significant and reliable improvement in shame-proneness and PTSD after one year of both tested therapies, DBT and DBT + DBT PE ^1^.
Held et al. (2015)	Treatment-seeking substance users (*n* = 72)	Cross-sectional	PCL-S	SSGS	Trauma-related shame provided direct and indirect (through avoidant coping) pathways for the relationship between trauma-related guilt and PTSD severity.
Hundt and Holohan (2012)	Treatment-seeking adult male veterans (*n* = 264)	Cross-sectional	PCL-C	ISS	Trait shame mediation of PTSD and IPV perpetration relationship not significant when depression taken into account.
La Bash and Papa (2014)	College students (*n* = 99)	Cross-sectional	PCL	Modified TLEQ	Peritraumatic shame mediated relationship between risk factors (trauma type and number of potentially traumatic events) and PTSD symptoms.
Layer et al. (2004)	Adult females (*n* = 35)	Longitudinal	IES-R	ISS	Spiritually-based group intervention associated with improvement in trait shame and PTSD symptoms in women suffering from post-abortion grief.
Leskela et al. (2002)	Adult male veterans (*n* = 107)	Cross-sectional	PCL-M and CES	TOSCA	Shame-proneness, not guilt-proneness, positively correlated with PTSD severity.
Lowinger and Solomon (2004)	Adult males convicted of reckless driving (*n* = 75)	Cross-sectional	PTSD-I	TOSCA	Trait shame did not significantly differ between reckless drivers who had caused an accidental death and control group.
Negrao II et al. (2005)	Female children and adults (*n* = 137)	Cross-sectional	PSS	EMFACS (nonverbal) and narrative coding	Coherence between facial and verbal shame expression correlated with PTSD severity in non-disclosing group of CSA survivors.
Ojserkis et al. (2014)	College students (*n* = 45)	Cross-sectional	LEC, PCL-C, IES-R, and PCI	TOSCA and visual analogue scale	State shame, but not trait shame, correlated with PTSS.
Øktedalen et al. (2015)	Norwegian adults in an inpatient setting (*n* = 65)	Longitudinal; RCT	PSS-I and PSS-SR	Study-designed self-report measure	Within-person improvements in trauma-related shame and trauma-related guilt predicted subsequent reductions in PTSD during both interventions, PE^1^ and modified PE.
Pineles et al. (2006)	College females (*n* = 156)	Cross-sectional	PCL	TOSCA	Shame-proneness, independent of guilt-proneness, predicted PTSD symptoms whereas guilt independent of shame did not.
Resick et al. (2008)	Adult female victims of interpersonal violence (*n* = 150)	Longitudinal; RCT	CAPS and PDS	ESS	PTSD and trait shame improved over time in all three conditions (components of CPT ^1^).
Robinaugh and McNally (2010)	Adults (*n* = 140)	Cross-sectional	PCL	SSGI	Event-related shame predicted PTSD symptoms.
Schoenleber et al. (2015)	Adult male survivors of interpersonal trauma (*n* = 103)	Cross-sectional	LEC and PCL-C	PANAS (single-item)	Trait shame accounted for the association between posttraumatic symptoms and aggressive behavior whereas trait guilt did not.
Semb et al. (2011)	Adult victims of single violent crime (*n* = 35)	Cross-sectional	HTQ	TOSCA and visual analogue scale	Both shame-proneness and event-related shame positively correlated with severity of posttraumatic distress. Level of event-related shame mediated the effect of shame-proneness on posttraumatic symptoms.
Shin et al. (2014)	Treatment-seeking Korean adult female survivors of sexual violence (*n* = 38)	Longitudinal; prospective	CAPS, PSS-SR, and PCI	PFQ	No correlation between shame-proneness and PTSD severity once depression severity was controlled. Similar findings for guilt-proneness.
Sippel and Marshall (2011)	Adult civilians (*n* = 47)	Cross-sectional	CAPS	Emotional Stroop task (implicit) and self-referential encoding task (implicit)	Speed of implicit shame processing and PTSD severity inversely correlated. Shame processing mediated relationship between PTSD severity and frequency of IPV perpetration.
Stotz et al. (2015)	Male refugee youth in Germany (age 11–20) (*n* = 32)	Cross-sectional	UCLA PTSD	SVQ	Both trauma-related shame and guilt positively correlated with PTSD severity.
Street and Arias (2001)	Adult female IPV survivors in domestic violence shelters (*n* = 63)	Cross-sectional	MS-Civilian	TOSCA	Shame-proneness significantly predicted of PTSD symptoms, guilt-proneness did not.
Uji et al. (2007)	Japanese college females (*n* = 172)	Cross-sectional	IES-R	ASSQ and AAI	Event-related shame directly predicted PTSD, whereas attribution style did not.
Vidal and Petrak (2007)	Adult female survivors of adult sexual assault (*n* = 25)	Cross-sectional	IES-R	ESS and study-designed measure	Shame positively correlated with traumatic stress.

^1^ BPD = Borderline Personality Disorder; CCT = Child Centered Therapy; CPT = Cognitive Processing Therapy; CSA = Child Sexual Abuse; DBT = Dialectical Behavior Therapy; DBT PE = DBT Prolonged Exposure; IPV = Intimate Partner Violence; PE = Prolonged Exposure; PTS = Posttraumatic Stress; PTSS = Posttraumatic stress symptoms; RCT = Randomized Clinical Trial; TF-CBT = Trauma-Focused Cognitive Behavioral Therapy. ^2^ AAI = Abuse Attribution Inventory; ARBQ = Abuse-Related Beliefs Questionnaire; ASSQ = Abuse Specific Shame Questionnaire; BIGSS = Body Image Guilt and Shame Scale; CAPS = Children’s Attributions and Perceptions Scale; CES = Combat Exposure Scale; CITES-R = Children’s Impact of Traumatic Events Scale-Revised; CoSS = Compass of Shame Scale; CPSS = Child PTSD Symptom Scale; EMFACS = Emotion Facial Action Coding System; ESS = Experience of Shame Scale; FSCRS = Forms of Self-Criticizing/Attacking and Self-Reassuring Scale; HTQ = Harvard Trauma Questionnaire; IAE = Impact of Abuse Events; ISS = Internalized Shame Scale; K-SADS-PL = Kiddie-Schedule for Affective Disorders and Schizophrenia-Present and Lifetime Version; MS-Civilian = Civilian Mississippi Scale for PTSD; NSES = National Stressful Events Survey; OAS = Other as Shamer Scale; PCL-M = PTSD Checklist-Military; PCL-S = PTSD Checklist-Specific; PCI = Post-traumatic Cognitions Inventory; PDS = Posttraumatic Diagnostic Scale; PFQ2 = Harder Personal Feelings Questionnaire; PFQ-2 = The Personal Feelings Questionnaire-2; PSS-I = PTSD Symptom Scale-Interview; PSS-SR = PTSD Symptom Scale-Self Rating; PTSD-I = Posttraumatic Stress Disorder Inventory; PTSD-RI = Posttraumatic Stress Disorder Reaction Index; RCMS = Revised Civilian Mississippi Scale; SBA = Survey of Body Areas; SPTSS = Screen for Posttraumatic Stress Symptoms; SSGI = State Shame and Guilt Inventory; SSGS = State Shame and Guilt Scale; SRC = Stress Reactions Checklist for Disorders of Extreme Stress; SVQ = Shame Vulnerability Questionnaire; TAQ = Trauma Appraisal Questionnaire; TEQ = Trauma Emotion Questionnaire; TSI = Trauma Symptom Inventory; TSQ = The Shame Questionnaire; UCLA PTSD = UCLA PTSD Reaction Index.
